# Advances in Detecting RNA Modifications Using Direct RNA Nanopore Sequencing

**DOI:** 10.1002/ggn2.202500041

**Published:** 2025-10-06

**Authors:** Yaran Liu, Yang Li, Qiang Sun

**Affiliations:** ^1^ Institute of Medical Artificial Intelligence Binzhou Medical College Yantai Shandong 264003 P. R. China; ^2^ Center for RNA Medicine the Fourth Affiliated Hospital of School of Medicine International School of Medicine International Institutes of Medicine Zhejiang University Yiwu Zhejiang 322000 P. R. China; ^3^ Genetic Diseases Key Laboratory of Sichuan Province Department of Medical Genetics Department of Laboratory Medicine Sichuan Academy of Medical Sciences & Sichuan Provincial People's Hospital School of Medicine University of Electronic Science and Technology of China Chengdu Sichuan 610072 P. R. China; ^4^ Sichuan‐Chongqing Joint Key Laboratory of Pathology and Laboratory Medicine Jinfeng Laboratory Chongqing 401329 P. R. China

**Keywords:** direct RNA sequencing, epitranscriptomics, Oxford Nanopore Technologies, RNA modifications, single‐molecule analysis

## Abstract

RNA modifications add a dynamic and versatile regulatory layer to gene expression, influencing RNA stability, splicing, translation, and cellular responses. Despite their importance, traditional detection methods—such as antibody‐based enrichment, chemical labeling, or indirect sequencing approaches—often suffer from limited resolution, biases, and an inability to capture modifications in their native RNA context. Oxford Nanopore Technologies (ONT) direct RNA sequencing (DRS) overcomes many of these limitations by enabling amplification‐free, single‐molecule, and single‐nucleotide detection of diverse RNA modifications directly from native RNA molecules. In this review, recent advances in applying ONT DRS to characterize modifications beyond the extensively studied N^6^‐methyladenosine (m^6^A), including 2′‐O‐methylation (Nm), N^1^‐methyladenosine (m^1^A), 5‐methylcytosine (m^5^C), N^4^‐acetylcytidine (ac^4^C), N^7^‐methylguanosine (m^7^G), pseudouridine (Ψ), and adenosine‐to‐inosine (A‐to‐I) editing are summarized. Computational frameworks and basecalling innovations are highlighted that improve modification calling, with particular emphasis on approaches that detect co‐occurring modifications and reveal their potential regulatory cross‐talk within individual transcripts. Finally, emerging applications across synthetic systems, non‐model organisms, and disease contexts are discussed, and offer a forward‐looking perspective on integrating nanopore‐based epitranscriptomics with multi‐omics platforms to achieve a deeper and more comprehensive understanding of RNA regulation.

## Graphical abstract

This review examines recent advances in Oxford Nanopore Technologies direct RNA sequencing, highlighting its expanding capacity to detect RNA modifications beyond m^6^A. It discusses computational frameworks and basecalling innovations that enable single‐nucleotide and single‐molecule resolution, explores co‐occurring modifications and their regulatory interactions, and surveys applications across synthetic systems, non‐model organisms, and disease contexts, offering a forward‐looking perspective on epitranscriptomics.

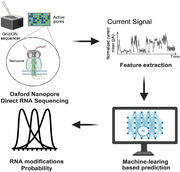

## 1 Introduction

**Figure 1:**
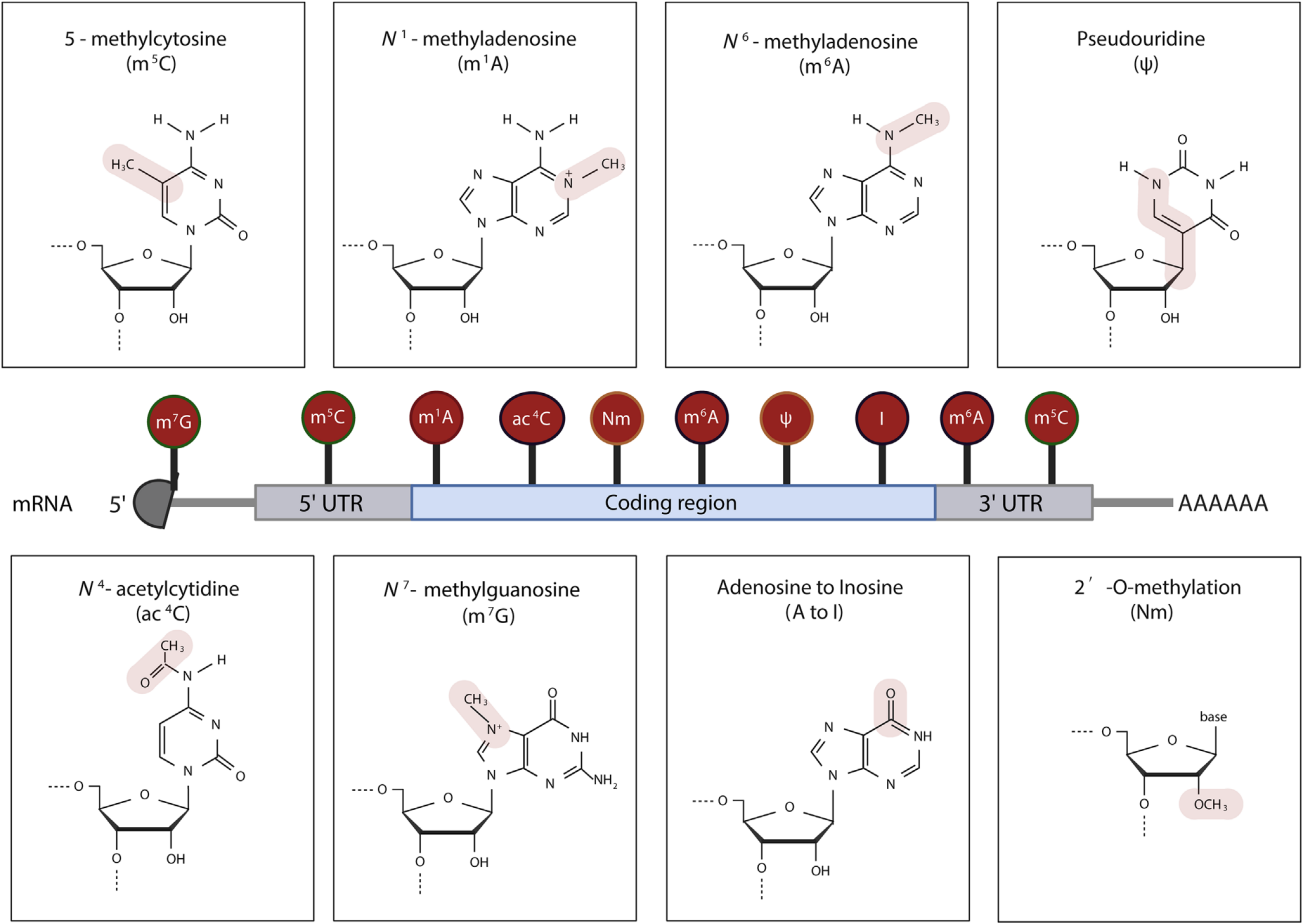
Diverse chemical modifications in eukaryotic mRNAs and their distribution across transcript regions. This schematic illustrates major RNA modifications detectable by Oxford Nanopore Technologies (ONT) direct RNA sequencing, including 5‐methylcytosine (m^5^C), N1‐methyladenosine (m^5^A), N6‐methyladenosine (m⁶A), pseudouridine (Ψ), N4‐acetylcytidine (ac^4^C), N7‐methylguanosine (m^7^G), adenosine‐to‐inosine editing (A‐to‐I), and 2′‐O‐methylation (Nm). Each panel displays the chemical structure of the modified nucleoside, with the altered functional group highlighted in pink. The central diagram depicts the typical localization of these modifications along the mRNA transcript, including the 5′ untranslated region (UTR), coding region, and 3′ UTR. The m⁷G cap is shown at the 5′ end, while other modifications are positioned based on known or representative enrichment patterns.

Post‐transcriptional biochemical modifications of RNA molecules constitute a critical layer of gene expression regulation, leading to the emerging field of epitranscriptomics.^[^ ^1^, ^2^ ^]^ More than 170 distinct chemical modifications have been identified across various types of RNA, ranging from messenger RNAs (mRNAs) to non‐coding RNAs (ncRNAs), such as ribosomal RNA (rRNA), transfer RNA (tRNA), and small nuclear RNA (snRNA).^[^ ^3^ ^]^ The prevalent RNA modifications including N6‐methyladenosine (m^6^A), 5‐methylcytidine (m^5^C), N4‐acetylcytidine(ac^4^C), 2′‐O‐methyl nucleoside (Nm), 7‐methylguanosine (m^7^G), inosine (I), pseudouridine (Ψ), N1‐methyladenosine (m^1^A), adenosine‐to‐inosine (A‐to‐I) editing, cytidine‐to‐uridine (C‐to‐U) editing, 5‐hydroxymethylcytidine(hm^5^C), and 5‐methoxyuridine(5moU), are found to affect several post‐transcriptional processes, such as pre‐mRNA splicing, nuclear export, mRNA decay, mRNA translation, RNA localization, and primary microRNA processing^[^ ^3^, ^4^, ^5^, ^6^, ^7^, ^8^ ^]^ (**Figure** **1**). RNA modifications are essential in multiple biological processes, encompassing nearly all aspects of RNA metabolism, fundamental cellular activities,^[^ ^9^ ^]^ organismal development,^[^ ^10^ ^]^ and physiological homeostasis.^[^ ^11^ ^]^ Importantly, their dysregulation is implicated in cancer pathogenesis^[^ ^12^, ^13^, ^14^, ^15^ ^]^ and various pathological conditions.^[^ ^16^, ^17^, ^18^ ^]^ Understanding the landscape and dynamics of RNA modifications is pivotal for decoding post‐transcriptional gene regulation. However, the identification and functional characterization of many RNA modifications, especially those present at low abundance or occurring transiently, remain challenging.

RNA modification detection methods have advanced the transcriptome‐wide mapping and functional investigations of RNA modifications.^[^ ^1^, ^19^ ^]^ The majority of current RNA modification detection approaches, including (1) quantification methods for detection of global RNA modification abundance, such as liquid chromatography–tandem mass spectrometry (LC‐MS/MS)^[^ ^20^ ^]^ and enzyme‐linked immunosorbent assay (ELISA)‐based methods^[^ ^21^ ^]^; (2) locus‐specific detection methods, such as single‐base elongation and ligation‐based qPCR amplification method (SELECT), and (3) transcriptome‐wide profiling approaches that combine next‐generation sequencing (NGS) with antibody‐based RNA immunoprecipitation or chemical derivatization techniques, such as methylated RNA immunoprecipitation sequencing (MeRIP‐seq),^[^ ^22^ ^]^ m^6^A RNA immunoprecipitation sequencing (m^6^A‐RIP‐seq),^[^ ^23^, ^24^, ^25^, ^26^, ^27^, ^28^, ^29^ ^]^ methylation individual‐nucleotide resolution crosslinking immunoprecipitation sequencing (miCLIP‐seq),^[^ ^30^ ^]^ and RNA bisulfite sequencing (RNA‐BisSeq and RBS‐Seq).^[^ ^31^ ^]^ These approaches have significantly advanced our understanding of the epitranscriptome. However, these methods are often constrained by key limitations,^[^ ^22^ ^]^ such as false positives arising from cross‐reactivity and low specificity of antibodies or chemical reactions, biases introduced by complex experimental procedures, a lack of single‐molecule resolution, and difficulty in detecting low‐abundance or isoform‐specific modifications. In recent years, experimental approaches relying on NGS have been widely applied for transcriptome‐wide RNA modification detection.^[^ ^23^, ^24^, ^25^, ^26^, ^27^, ^28^, ^29^ ^]^ However, these methods are limited by several factors, including the sequencing length (typically 50–300 bp), the requirement for reverse transcription of RNA into complementary DNA (cDNA), which can erase native RNA modifications, and the restricted capacity for simultaneous detection of diverse RNA modification types.^[^ ^19^, ^30^ ^]^

Third‐generation sequencing (TGS) technologies have emerged as powerful tools in transcriptomic research,^[^ ^31^ ^]^ and offer unprecedented capabilities in epitranscriptomic studies.^[^ ^32^ ^]^ Nanopore direct RNA sequencing (DRS) technology, developed by Oxford Nanopore Technologies (ONT), enables sequencing of native RNA molecules without the need for reverse transcription or amplification steps,^[^ ^33^ ^]^ thereby preserving the original epitranscriptomic modifications. When combined with computational approaches, DRS technology allows for the detection of RNA modifications at single‐molecule resolution,^[^ ^19^, ^22^ ^]^ including those present at low abundance.

In this review, we summarize the principles and recent applications of nanopore direct RNA sequencing technology for detecting diverse RNA modifications. Particular attention has been given to RNA modifications beyond m^6^A—such as Nm, m^1^A, m^5^C, ac^4^C, m^7^G, Ψ, and I—which remain less explored but are increasingly recognized as functionally important. Furthermore, we discuss key technical challenges in identifying these modifications, including their low abundance, signal overlap, and the need for advanced computational methods to analyze complex DRS data.^[^ ^30^ ^]^ Finally, we highlight emerging biological insights into RNA modifications and their potential roles in disease research, therapeutics, and biomarker discovery. Finally, we outline future directions for integrating ONT‐based sequencing with other omics technologies to achieve a comprehensive understanding of the epitranscriptome.

## 2 ONT Direct RNA Sequencing: A Tool for Uncharted Modifications

### 2.1 RNA Modifications: An Overview

The central dogma of molecular biology outlines the directional flow of genetic information from DNA to RNA to protein.^[^ ^34^ ^]^ Beyond transcriptional and translational control, post‐transcriptional RNA modifications have emerged as pivotal layers of gene regulation. RNA modifications are chemical alterations to RNA nucleotides that do not change the underlying sequence, which are collectively referred to as the “epitranscriptome”. These modifications play critical roles in regulating RNA structure and metabolism, including mRNA stability, translation, splicing, and export.^[^ ^35^ ^]^ To date, over 170 distinct RNA modifications have been identified across various types of RNA species, including mRNAs and non‐coding RNAs (rRNAs, tRNAs, snRNAs, and lncRNAs).^[^ ^3^ ^]^

Among these modifications, m^6^A, formed by methylation at the nitrogen‐6 position of adenosine, is the most abundant internal modification in eukaryotic mRNAs. It plays critical roles in regulating mRNA stability, splicing and export, thereby influencing key cellular processes.^[^ ^6^ ^]^ It is deposited by a methyltransferase complex comprising METTL3, METTL14, and WTAP, and can be removed by demethylases such as FTO and ALKBH5.^[^ ^36^, ^37^, ^38^ ^]^ m^5^C, originally known as a DNA modification, also occurs at low levels in eukaryotic mRNAs. It refers to the methylation of the fifth carbon of cytosine in RNA nucleotides. Although m^5^C represents a small fraction of total cytidines in mRNAs, it plays important regulatory roles, particularly in mRNA stabilization and export.^[^ ^39^ ^]^ ac^4^C is a conserved RNA modification catalyzed by NAT10.^[^ ^40^ ^]^ It was initially characterized in tRNAs and rRNAs but has more recently been identified in human mRNAs. In coding sequences, ac^4^C enhances translation efficiency by stabilizing codon‐anticodon interactions, whereas its presence in 5′ UTRs may inhibit translation initiation.^[^ ^41^, ^42^ ^]^ Nm is a widespread RNA modification present at both cap‐adjacent and internal positions of mRNAs, where it influences mRNA stability, translation, and splicing.^[^ ^43^, ^44^ ^]^ Internal Nm can inhibit translation elongation and modulate splicing, whereas cap‐adjacent Nm (Cap‐Nm) serves as a self‐recognition marker to evade innate immune sensing.^[^ ^45^, ^46^ ^]^

m^7^G is a critical RNA modification located at the 5′ cap of mRNAs and internal sites of tRNAs, rRNAs, and some miRNAs, playing essential roles in mRNA stability, translation initiation, and nuclear export. Its deposition is mediated by writer complexes such as METTL1/WDR4 in tRNAs and RNMT/RAM in mRNA caps, while QKI proteins have been identified as m^7^G readers.^[^ ^47^, ^48^ ^]^ Dysregulation of m^7^G methyltransferases leads to aberrant m^7^G methylation and contributes to cancer initiation and progression by affecting gene expression and oncogenic pathways.^[^ ^47^, ^49^ ^]^ m^1^A is a conserved RNA modification at the N^1^ position of adenosine that occurs in tRNAs, mRNAs, rRNAs, and lncRNAs. It influences RNA structure and translation.^[^ ^50^ ^]^ The aberrant activity of its dedicated writers, erasers, and readers is increasingly linked to oncogenic proliferation, metabolic reprogramming, and drug resistance, positioning the m^1^A machinery as a promising source of both biomarkers and therapeutic targets.^[^ ^51^, ^52^ ^]^

Inosine, generated via A‐to‐I RNA editing by ADAR enzymes, introduces transcriptomic diversity by modulating splicing, mRNA stability, and protein function.^[^ ^53^ ^]^ Inosine alters splicing by modifying splice sites and regulates mRNA stability through interactions with miRNAs and RNA‐binding proteins. These regulatory mechanisms play essential roles in brain development, immune regulation, and disease pathogenesis.^[^ ^53^, ^54^, ^55^ ^]^ Ψ is catalyzed by multiple pseudouridine synthases and is widely distributed in mRNAs. It influences translation read‐through, alternative splicing, and transcript stability in a context‐dependent manner.^[^ ^56^ ^]^

Like mammals, m^6^A is the most abundant internal modification in plant mRNAs. It is deposited by a conserved writer complex containing MTA, MTB, VIR, FIP37, and HAKAI, and can be dynamically removed by ALKBH10B and ALKBH9B.^[^ ^57^ ^]^ m^6^A regulates various aspects of mRNA metabolism, including stability, alternative polyadenylation, secondary structure, and translation efficiency, and plays essential roles in biological processes such as embryogenesis, meristem maintenance, floral transition, and responses to environmental stress.^[^ ^57^ ^]^ Ψ is widely distributed across different plant RNAs. It is catalyzed by pseudouridine synthases, including RluA, RsuA, TruA, TruB, PUS10, and TruD.^[^ ^58^ ^]^ Ψ enhances RNA secondary structure and modulates RNA‐protein interactions, thereby impacting ribosome biogenesis, splicing fidelity, and plant stress responses in *Arabidopsis thaliana*.^[^ ^59^ ^]^ m^5^C is present in diverse RNA species, including mRNAs, tRNAs, rRNAs, and non‐coding RNAs in *Arabidopsis thaliana* and *Oryza sativa*, and is catalyzed by conserved methyltransferases such as TRM4B, NSUN2, NSUN5, and TRDMT1.^[^ ^60^, ^61^ ^]^ m^5^C enhances transcript stability to promote translation efficiency, and facilitates long‐distance RNA transport, particularly for mobile mRNAs critical for inter‐organ communication.^[^ ^61^, ^62^ ^]^ Dynamic m^1^A RNA methylation is widespread in mRNAs, lncRNAs, and circular RNAs (circRNAs), and acts as a key epitranscriptomic regulator of fruit ripening in tomato.^[^ ^63^, ^64^ ^]^ ac^4^C has recently emerged as a conserved epitranscriptomic marker in angiosperms. It is enriched in coding sequences, particularly near translation start sites, suggesting potential roles in RNA stability, splicing, and translation.^[^ ^65^ ^]^ In plants, three homologs of the human NAT10 acetyltransferase are responsible for catalyzing the N^4^‐acetylation of cytidine (ACYR1/ACYR2 in Arabidopsis^[^ ^66^ ^]^ and OsNAT10 in rice^[^ ^67^ ^]^). Loss‐of‐function mutants in these genes dramatically reduce ac^4^C levels, and lead to developmental defects, highlighting the essential roles.^[^ ^67^, ^68^ ^]^ Functionally, ac^4^C promotes the translation of specific transcripts in *Arabidopsis thaliana* and *Oryza sativa*, such as members of the light‐harvesting complex (LHC) gene family (*LHCB2*, *LHCB3*, *LHCB4*), and the OsLIR1, a key regulator of photosynthetic electron transfer.^[^ ^67^, ^68^ ^]^ In response to pathogen infection, OsNAT10 acetylates *OsAOC* mRNA, which encodes a core enzyme in jasmonic acid biosynthesis, thereby boosting its translation and rapidly activating jasmonate‐mediated immune responses.^[^ ^69^ ^]^ Nm and m^7^G have also been detected in *Arabidopsis thaliana* rRNAs, but their functions remain unclear.^[^ ^70^, ^71^ ^]^

### 2.2 Current Detection Methods and Limitations

Over the past decade, multiple methodologies have been developed to detect RNA modifications across the transcriptome, each with distinct principles and limitations. Current sequencing strategies for RNA modification detection are commonly classified into four major categories.^[^ ^72^ ^]^ Antibody‐based methods, which are widely adopted for profiling marks such as m^6^A, m^5^C, m^7^G, and ac^4^C, depend on modification‐specific immunoprecipitation.^[^ ^40^, ^48^, ^62^, ^73^, ^74^ ^]^ However, these approaches typically require large amounts of RNA input and often suffer from limited specificity, batch variability, and lack stoichiometric resolution.^[^ ^22^ ^]^ Reverse transcription signature‐dependent techniques detect RNA modifications through their impact on cDNA synthesis, including the induction of mismatches, premature terminations, or insertions and deletions at modification sites.^[^ ^75^, ^76^ ^]^ Although relatively straightforward and cost‐effective, these methods are susceptible to false positives due to sequence polymorphisms, sequencing artifacts, and library preparation biases.^[^ ^19^ ^]^ Enzyme‐dependent methods utilize the substrate recognition or cleavage specificity of certain demethylases, endonucleases, or exonucleases to distinguish modified from unmodified nucleotides. These methods are constrained by enzymatic efficiency and context‐dependent sequence preferences. Finally, chemical‐assisted approaches combine selective chemical labeling or derivatization of modified bases with next‐generation sequencing (NGS). These strategies have recently enabled base‐resolution identification and quantification of marks such as m^6^A, Ψ, m^5^C, and ac^4^C.^[^ ^77^ ^]^ Nonetheless, the effectiveness of these methods hinges on reaction conditions that must preserve RNA integrity, which remains a technical challenge.

### 2.3 Principles of ONT Sequencing

**Figure 2:**
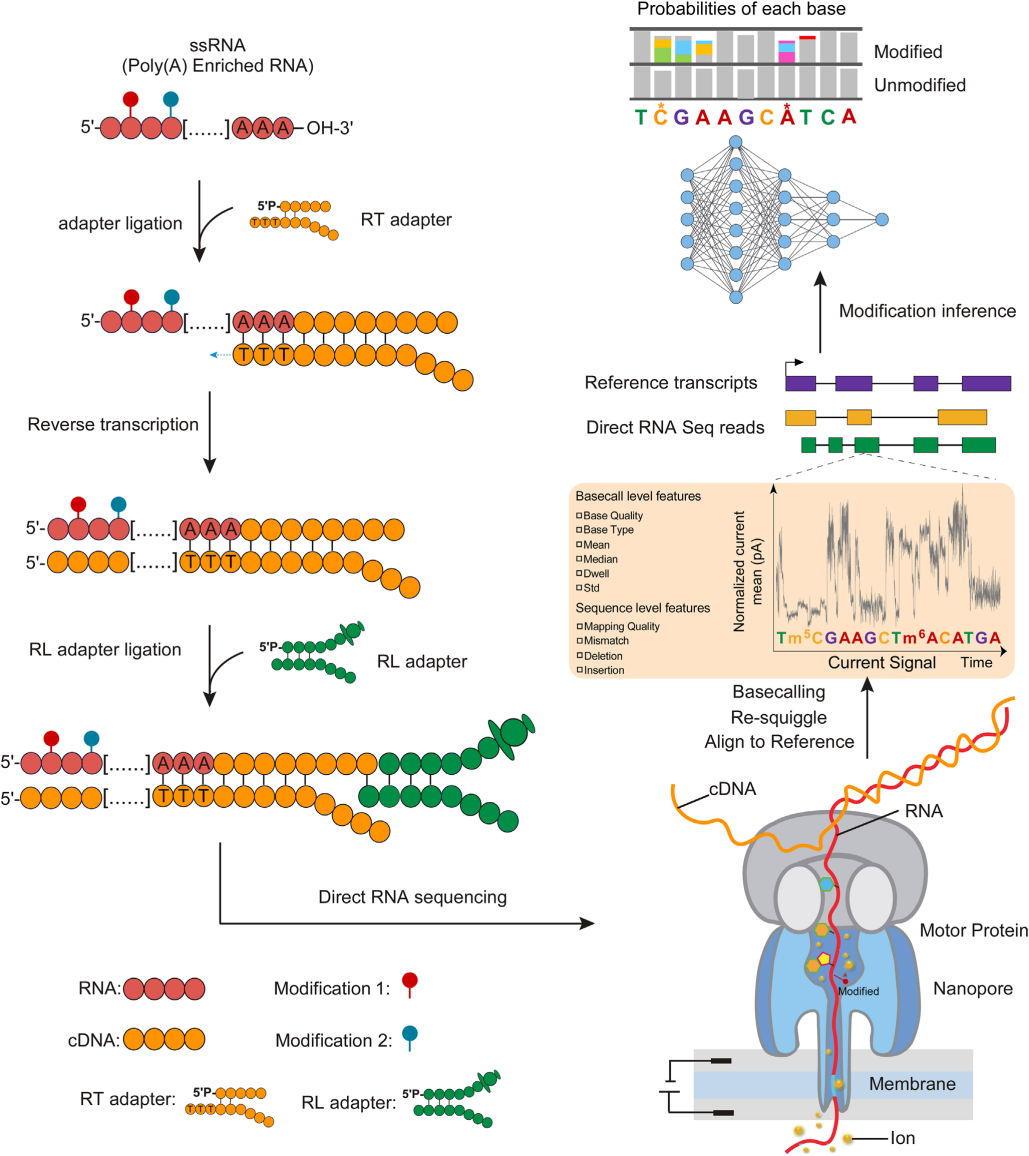
Schematic overview of the Oxford Nanopore Technologies (ONT) direct RNA sequencing workflow and RNA modification detection. Schematic overview of the ONT DRS workflow and RNA modification detection. Poly(A)‐enriched single‐stranded RNA (ssRNA) is ligated to a reverse transcription (RT) adapter at the 3′ end, followed by reverse transcription to generate complementary DNA (cDNA). A sequencing (RL) adapter is then ligated to the cDNA‐RNA hybrid, enabling direct RNA sequencing on a nanopore platform. As RNA passes through the nanopore, an associated motor protein regulates the translocation speed, and ionic current changes are measured in real time. RNA modifications alter the local ionic current signal, which is recorded along with the nucleotide sequence. Computational analysis involves basecalling, signal re‐squiggling, and alignment to a reference transcriptome, followed by feature extraction and machine learning–based prediction to infer the modification type and position.

Nanopore sequencing is a real‐time, single‐molecule sequencing platform that determines nucleic acid sequences by measuring changes in ionic current as individual RNA or DNA molecules translocate through a nanopore embedded in an electrically resistant membrane^[^ ^78^ ^]^ (**Figure** **2**). An applied voltage generates an electrochemical gradient that drives nucleic acids through a biological pore,^[^ ^79^ ^]^ while a processive motor protein unwinds and ratchets the strand through the pore at a controlled speed. As the molecule moves forward through the pore, specific combinations of nucleotides (typically 5‐mers) occupy the pore's constriction site and induce characteristic disruptions in the ionic current.^[^ ^80^ ^]^ These current signals are recorded with high temporal resolution and decoded into nucleotide sequences using traditional statistical methods (such as hidden Markov models) or machine learning (ML) methods (such as deep neural networks).^[^ ^80^, ^81^ ^]^

In DRS, native RNA molecules are sequenced directly without reverse transcription or amplification, thereby preserving endogenous modifications and avoiding cDNA synthesis biases. Each read retains both sequence and modification‐sensitive signal features, making it possible to analyze transcriptomes while simultaneously capturing information about RNA modifications.^[^ ^78^, ^80^ ^]^ This fundamental principle forms the basis for the method's growing utility in epitranscriptomics, enabling a more direct and comprehensive investigation of the transcriptome landscape than conventional NGS approaches.

## 3 Applications of ONT Sequencing in Detecting Modifications

Accurate detection of RNA modification sites and their levels across biological samples is essential for understanding their functional roles under various conditions. However, most current profiling methods based on NGS technologies for transcriptome‐wide RNA modification profiling typically require reverse transcription, a process that leads to the loss of native modification signals from RNA molecules, such as antibody enrichment or chemical treatment.^[^ ^80^ ^]^ In addition, these enrichment‐based strategies suffer from several limitations, including PCR‐induced bias, limited specificity of antibodies or chemical reagents, and the inability to achieve single‐molecule resolution.^[^ ^72^ ^]^ TGS technologies, particularly ONT DRS, have emerged as powerful alternatives, enabling the direct interrogation of native RNA molecules and the detection of modifications with single‐molecule resolution.^[^ ^33^ ^]^ Unlike traditional approaches, these technologies allow the identification of RNA modifications without the need for reverse transcription or amplification. While DRS enables direct sequencing of native RNA molecules, accurately identifying modification‐induced signal perturbations remains a technically demanding task owing to the complexity and variability of raw signal data.^[^ ^31^ ^]^ To address these challenges, a growing number of computational tools have been developed for the analysis of RNA modifications from DRS data.

Recent updates in ONT DRS have significantly increased the platform's accuracy, sensitivity, and utility for RNA modification detection. The transition from RNA002 to RNA004 chemistry introduced a new RNA‐specific nanopore and was accompanied by an updated protocol featuring more efficient reverse transcriptase and reduced RNA input requirements. These improvements facilitate broader application of direct RNA sequencing, especially in studies where RNA quantity is limited.^[^ ^82^, ^83^, ^84^ ^]^

Comparative analyses using data generated from the same RNA sample demonstrated substantial performance gains. RNA004 chemistry produced a greater number of aligned reads and achieved higher sequencing accuracy, with median alignment identity increasing from 90.65% (RNA002) to 98.67% (RNA004). In addition, RNA004 exhibited a threefold reduction in base substitution rates and a pronounced decrease in insertion/deletion (INDEL) rates, from 7.19% to 0.88%, reflecting improved basecalling precision and read integrity. These advances directly impact RNA modification detection. Legacy approaches such as U‐to‐C mismatch‐based Ψ identification, developed under RNA002, showed limited concordance with the RNA004 data. In contrast, the Dorado basecaller introduced with RNA004, leveraging ionic current signals rather than mismatch patterns, enabled more accurate de novo detection of RNA modifications, including Ψ, m^6^A, m^5^C, and inosine. Validation using synthetic oligonucleotides confirmed high model performance, with accuracy and F1‐scores exceeding 94% for both Ψ and m^6^A.^[^ ^82^ ^]^

Overall, RNA004 chemistry represents a major advancement in nanopore‐based DRS. Lower RNA input requirements, and improved accuracy position this platform as a powerful tool for transcriptome‐wide epitranscriptomic profiling across a wider range of biological contexts.

### 3.1 Computational Methods for Detecting m^6^A Modification

**Figure 3:**
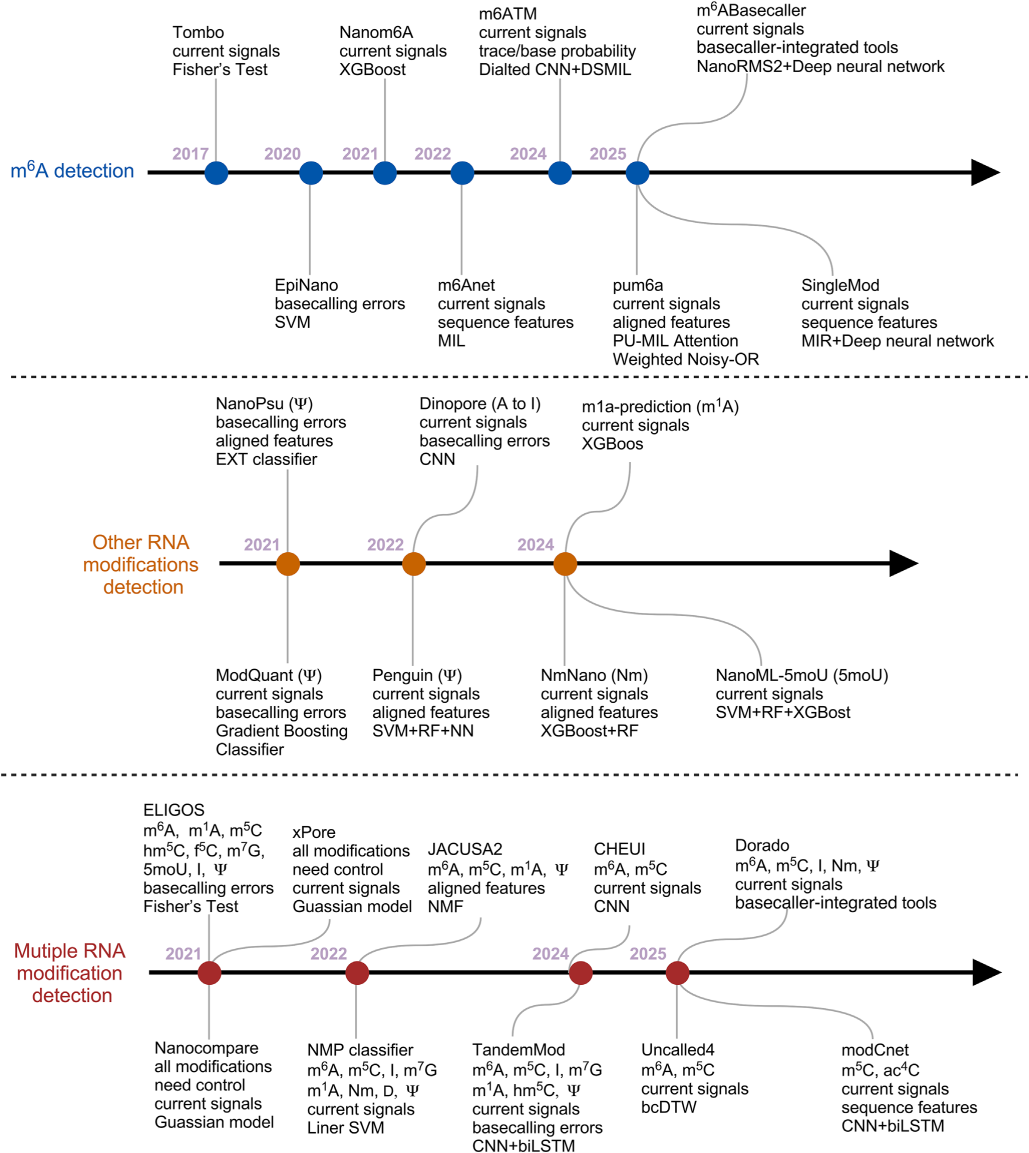
At the timeline of computational tools for ONT RNA modification detection. Chronological overview of major computational tools developed for RNA modification detection, which are grouped into three categories: m^6^A detection (blue), detection of other individual RNA modifications (orange), and multi‐modification detection (red). Each entry lists the primary input features used and the main computational framework or algorithm. The timeline illustrates the methodological progression from early statistical tests and classical machine learning to modern deep learning and real‐time, basecaller‐integrated approaches.

To systematically review recent advances, we first discuss computational algorithms for detecting m^6^A modifications from DRS data (**Table** **1**). The core challenge tackled by this diverse and rapidly evolving toolkit is deciphering the subtle signal perturbations caused by m^6^A. Consequently, these methods employ a wide spectrum of computational strategies ranging from statistical tests to sophisticated deep learning architectures, leveraging different signal features to achieve objectives such as site detection or stoichiometry quantification. The following sections provide a detailed exposition of the methodologies, underlying principles, and key innovations that define these significant computational frameworks (**Figure** **3**).

**Table 1:** Comparative summary of computational tools for m^6^A profiling from Nanopore DRS data.

Methods	Computational algorithm	Detection features	Application	Limitations	AUC	Year	Refs.
Tombo	Fisher's test	Current signals	*H. sapiens*	Control	0.62‐0.97	2017	[85]
DiffErr	G‐test	Base‐calling error	*A. thaliana*	Control	–	2020	[86]
DRUMMER	G‐test	Current signals	*H. sapiens*	Control	–	2020	[87]
MINES	RF	Current signals	*H. sapiens*	Control/Motif	0.54–0.76	2020	[88]
EpiNano	SVM	Base‐calling error	*S. cerevisiae*	Control/Motif	0.70–0.94	2020	[89]
Nanom6A	XGBoost	Current signals	*H. sapiens* *P. trichocarpa*	Control/Motif	0.97	2021	[90]
Yanocomp	GMM	Current signals	*A. thaliana*	Control	–	2021	[92]
DENA	Bi‐LSTM	Current signals	*H. sapiens* *A. thaliana*	Control/Motif	0.90–0.97	2022	[93]
m6Anet	MIL	Current signals, sequence features	*H. sapiens* *A. thaliana*	Motif	0.83–0.90	2022	[94]
m6ATM	Dilated CNN+ DSMIL	Current signals, base‐calling error	*H. sapiens*	Control/Motif	0.87– 0.97	2024	[95]
RedNano	ResNet	Current signals, base‐calling error	*H. sapiens* *A. thaliana* *P. trichocarpa*	Motif	0.75–0.99	2024	[96]
Xron	CRNN + NHMM	Current signals	*H. sapiens*	–	0.83–0.99	2024	[97]
pum6a	PU‐MIL + Weighted Noisy‐OR	Current signals, alignment features	*H. sapiens*; *M. musculus*	–	0.81–0.95	2025	[98]
m6Aiso	2D‐ResNet	Current signals, sequence features	*H. sapiens*	Control/Motif	0.9	2025	[99]
SingleMod	DNN+ MIR	Current signals, sequence features	*H. sapiens*	Motif	0.95–0.98	2025	[100]
m^6^ABasecaller	NanoRMS2+ DNN	Current signals	*H. sapiens* *M. musculus* *D. rerio*	Control	–	2025	[101]
DEMINERS	DecodeR+ Densecall	Current signals	Multiple	–	0.99	2025	[102]

#### 3.1.1 Signal‐Level and Statistical Approaches

Early computational frameworks, such as Tombo, DiffErr, and DRUMMER, focused on detecting m^6^A by analyzing deviations in raw signals or base‐calling error rates. These methods employ statistical comparisons between experimental conditions (e.g., wild‐type versus knockout lines) or expected signal models to identify modified nucleotides. While conceptually simple, these tools lay the foundation for more sophisticated approaches and remain valuable for initial site detection and hypothesis generation.

Tombo is a widely used computational framework designed for detecting DNA and RNA modifications directly from nanopore sequencing data. It improves upon earlier signal‐level analysis tools by implementing a refined signal re‐alignment procedure (“resquiggling”) and statistical testing to compare observed signals with expected models or control samples.^[^ ^85^ ^]^ Tombo supports both model‐based and non‐model‐based approaches, allowing the detection of modifications without requiring specialized sample preparation. Its flexibility and integration with Oxford Nanopore's output have made it a standard pipeline for epigenetic and epitranscriptomic analyses.

Parker et al. proposed differential error rate analysis (DiffErr), a method that detects m^6^A modifications by comparing base‐calling error profiles between wild‐type and m^6^A‐deficient *Arabidopsis* using nanopore DRS data.^[^ ^86^ ^]^ This approach requires neither retraining nor antibody enrichment and shows high concordance with miCLIP. This represents a key innovation in the use of native sequencing errors as proxies for RNA modification detection.

DRUMMER is a computational algorithm that utilizes DRS data to detect m^6^A modifications at single‐nucleotide resolution by comparing base‐calling error rates between *METTL3* knockout and wild‐type conditions.^[^ ^87^ ^]^ By integrating meRIP‐seq with DRS, DRUMMER maps m^6^A sites across the adenoviral transcriptome, and demonstrates that m^6^A plays a critical role in regulating the splicing efficiency of viral transcripts, providing new insights into RNA modification during viral infection.

#### 3.1.2 Classic Machine Learning Approaches

Classical machine learning methods such as EpiNano, MINES, Nanom6A, and Yanocomp integrate engineered signal features, base‐calling error profiles, and sequence motif filters (e.g., DRACH, a sequence motif defined as D = A/G/U, R = A/G, H = A/C/U, with the canonical m^6^A motif being DRACH) with supervised learning frameworks. Some of these tools incorporate orthogonal datasets (e.g., CLIP‐seq and MeRIP‐seq) to improve accuracy. They enable isoform‐resolved m^6^A mapping and demonstrate the utility of combining multiple feature types for robust site detection, although their resolution is often limited by training data and motif constraints.

MINES is a random forest (RF)‐based tool that enables base‐specific identification of m^6^A modifications from DRS data.^[^ ^88^ ^]^ By integrating nanopore signal features with DRACH motif filtering and supervised learning using CLIP‐seq validated sites, MINES enables isoform‐resolved m^6^A mapping in human transcriptomes. Experimental validation involving METTL3 knockdown and ALKBH5 overexpression further supported the specificity and biological relevance of the predictions.

Liu et al. developed EpiNano, a machine learning (ML) framework that identifies m^6^A RNA modifications in DRS data by exploiting reproducible base‐calling errors.^[^ ^89^ ^]^ When trained on synthetic constructs, the model distinguishes modified sites from unmodified sites using features such as mismatch frequency and base quality. This approach circumvents the need for raw signal alignment and provides a computationally efficient method for epitranscriptomic profiling.

Nanom6A is an ML‐based pipeline that quantifies m^6^A modifications at single‐nucleotide resolution from nanopore DRS signals using an XGBoost classifier trained on engineered signal features derived from RRACH motifs.^[^ ^90^ ^]^ The method achieves high accuracy and reproducibility, validated by MeRIP‐Seq, m^6^A‐REF‐seq, and SCARLET assays across human, *Arabidopsis*, and *Populus* datasets. Recently, it has been combined with the LAFITE framework to enable isoform‐level m^6^A mapping from high‐confidence full‐length transcripts, providing new insights into the role of m^6^A in isoform‐specific regulation and subcellular localization.^[^ ^91^ ^]^

Yanocomp is a robust computational tool for identifying m^6^A modifications in nanopore DRS data using multivariate Gaussian mixture models (GMMs) augmented with a uniform component to handle outliers.^[^ ^92^ ^]^ By modeling adjacent *k*‐mers and supporting replicate‐aware comparisons, Yanocomp enables both differential modification detection and single‐molecule resolution analysis. The method is validated in *Arabidopsis* using vir‐1 and VIRc lines, in which known motifs and modification patterns are recovered.

#### 3.1.3 Deep Learning and Multiple‐Instance Learning (MIL) Frameworks

Recent advances increasingly rely on deep neural architectures to improve sensitivity, resolution, and generalizability. Tools such as m6Anet, DENA, m6ATM, RedNano, and Xron leverage architectures like Bidirectional Long Short‐Term Memory (Bi‐LSTM), WaveNet encoders, and convolutional‐recurrent networks to model complex relationships in raw ionic current signals. A notable trend is the adoption of MIL in models such as m6Anet, which overcomes the challenge of low or uneven modification coverage by aggregating read‐level predictions, allowing site‐level detection without per‐read supervision.

DENA (Deep‐learning Explore Nanopore m^6^A) is a Bi‐LSTM‐based model that detects and quantifies m^6^A at single‐nucleotide resolution from nanopore DRS data.^[^ ^93^ ^]^ It uniquely leverages in vivo transcribed RNA for training, using raw current signal features (mean, standard deviation, dwell time, and base quality) to overcome the limitations of models trained on synthetic RNAs. DENA enables isoform‐level m^6^A profiling with superior accuracy and cross‐species robustness.

m6Anet is a deep learning (DL) framework based on MIL to detect m^6^A modifications from DRS data.^[^ ^94^ ^]^ It uniquely addresses the lack of read‐level labels by aggregating single‐read predictions, thereby enabling accurate, single‐sample m^6^A identification and stoichiometry estimation. The method generalizes across cell types and species without retraining, and outperforms existing tools in both accuracy and robustness.

m6ATM is a DL framework for transcriptome‐wide m^6^A detection using nanopore DRS data.^[^ ^95^ ^]^ It employs a WaveNet‐based encoder to extract informative features from raw ionic current signals and alignment trace data, which are then analyzed using a dual‐stream MIL (DSMIL) architecture for site‐level modification prediction. The model demonstrates high predictive performance, achieving an area under the curve (AUC) of 0.923, particularly under conditions of low modification stoichiometry. Validation on both synthetic in vitro transcribed RNA and human cell line datasets confirmed its sensitivity and specificity, and its application revealed previously uncharacterized m^6^A‐associated regulatory pathways in liver cancer. However, its utility is currently constrained to DRACH motifs and relies on high sequencing coverage for accurate detection.

RedNano is a DL tool for m^6^A detection from Nanopore DRS data, leveraging dual‐modality features (raw current signals and base‐calling errors) processed through residual networks.^[^ ^96^ ^]^ It integrates dual‐modality features, including raw ionic current signals and base‐calling error profiles processed through a residual neural network architecture. By training on a diverse set of datasets, including synthetic transcripts, *Arabidopsis thaliana*, and human RNA, RedNano demonstrates strong cross‐species generalizability, with an AUC of 0.9405. The model outperforms existing approaches in independent validation using *Populus trichocarpa* data, highlighting its robustness across phylogenetically distant species. Nonetheless, its current implementation requires signal re‐alignment (resquiggling) and is restricted to detecting m^6^A within DRACH or RRACH sequence contexts.

Xron is a semi‐supervised DL framework for detecting m^6^A RNA modifications from ONT DRS data.^[^ ^97^ ^]^ It combines a convolutional recurrent neural network (CRNN) with a novel nonhomogeneous hidden Markov model (NHMM) to perform methylation‐aware base‐calling, thereby enabling simultaneous sequence decoding and modification detection. Xron demonstrates high predictive accuracy at both the read and site levels across synthetic, *Saccharomyces cerevisiae*, and human transcriptomes. Its hybrid training approach, leveraging in vitro transcribed data for augmentation and m^6^A immunoprecipitation data for fine‐tuning, enables robust and generalizable detection, even under low sequencing coverage conditions.

#### 3.1.4 Single‐Molecule and Low‐Coverage Sensitivity

Several DL frameworks extend detection beyond site‐level resolution, achieving single‐molecule sensitivity and robust quantification. Pum6a, SingleMod, and m6Aiso employ label‐efficient or semi‐supervised training strategies to handle low stoichiometry and limited read coverage. These approaches combine raw signal features, attention mechanisms, and advanced instance aggregation techniques, enabling precise quantification of m6A heterogeneity across molecules and transcript isoforms.

Pum6a represents a significant advancement in the detection of m^6^A from DRS data.^[^ ^98^ ^]^ To address the key limitations of earlier models relying on incomplete labels and high read coverage, pum6a introduces a positive‐unlabeled MIL (PU‐MIL) framework that integrates both raw electrical signal features and alignment‐derived metrics. Central to its architecture are a weighted Noisy‐OR aggregation function and an attention‐based instance selection module, which together enhance sensitivity and robustness, particularly at low‐coverage sites that are frequently missed by conventional approaches. In terms of strong performance across human, mouse, and cancer cell line datasets, Pum6a offers a technically innovative and generalizable solution, establishing it as one of the most refined m^6^A detection tools for DRS data.

m6Aiso is a DL framework for detecting m^6^A modifications at single‐molecule resolution from nanopore DRS data.^[^ ^99^ ^]^ The model is trained using endogenously labeled reads generated by APOBEC1‐YTH fusion protein–mediated C‐to‐U deamination, which marks m^6^A sites via the selective binding of the YTH domain. m6Aiso combines a two‐dimensional residual neural network (2D‐ResNet) with a semi‐supervised data‐cleaning strategy to minimize training noise and enhance model reliability. This approach enables accurate detection and quantification of both highly and sparsely methylated m^6^A sites, offering a valuable tool for transcriptome‐wide, single‐molecule m^6^A profiling.

SingleMod is a novel DL framework using multiple instance regression (MIR) to achieve precise quantification of m^6^A modifications at single‐molecule resolution from direct RNA sequencing data.^[^ ^100^ ^]^ By integrating data from multiple species and employing raw current signals as inputs, SingleMod provides robust and generalizable predictions of m^6^A heterogeneity and distribution patterns across diverse eukaryotes. This method significantly advances the field by enabling comprehensive epitranscriptome profiling beyond site‐level resolution.

#### 3.1.5 Basecaller‐Integrated and High‐Throughput Pipelines

A newer class of methods integrates m^6^A detection directly into base‐calling workflows, streamlining analysis and enabling real‐time modification detection. m6ABasecaller and DEMINERS exemplify this trend, combining base‐calling with per‐read modification probabilities or multiplexed analysis pipelines. These frameworks support high‐throughput applications, reduce computational overhead, and improve sensitivity for low‐stoichiometry modifications.

m6ABasecaller is a base‐calling model designed to be sensitive to RNA modifications, integrating m^6^A detection directly into the nanopore sequencing base‐calling workflow.^[^ ^101^ ^]^ The production of per‐read modification probabilities (modProb), enabling real‐time identification of m^6^A at single‐nucleotide and single‐molecule resolutions, without the need for genetic knockouts, read coverage thresholds, or post hoc processing. Compared with existing methods, which are trained on in vivo labeled data generated via NanoRMS2, m6ABasecaller exhibits notable improvements in sensitivity, specificity, and false positive rates, particularly in detecting low‐stoichiometry m^6^A sites. This integrated design streamlines the modification‐calling process and enhances the accessibility of high‐resolution m^6^A profiling in native transcriptomes.

DEMINERS is an integrated toolkit for DRS that enhances both data throughput and analytical resolution through two core components: DecodeR, an RF‐based demultiplexing module, and Densecall, a base‐caller inspired by DenseNet convolutional neural networks (CNNs).^[^ ^102^ ^]^ DEMINERS supports up to 24 multiplexed samples, incorporates species‐specific base‐calling, and enables RNA modification detection within a unified workflow. Compared with existing approaches, it demonstrates substantial improvements in precision, cost‐efficiency, and scalability, making it particularly well‐suited for high‐throughput transcriptomic and metagenomic studies using low‐input RNA samples.

A growing array of computational methods have emerged to detect m^6^A modifications from DRS data, underscoring the rapid evolution of epitranscriptomic informatics. These approaches span a diverse methodological spectrum from early statistical models such as Tombo, DiffErr, and DRUMMER to increasingly sophisticated DL frameworks. Classical machine learning tools, including EpiNano, MINES, and Nanom6A, typically rely on base‐calling error profiles or engineered features derived from ionic current signals, often incorporating motif filters or external orthogonal datasets such as CLIP‐seq or MeRIP‐seq to increase detection accuracy.

More recent advances are driven by deep neural architectures that leverage raw signal data and modern learning paradigms to improve both site‐level and isoform‐specific resolution. Notable examples include m6Anet, DENA, m6ATM, RedNano, and Xron, which employ advanced models such as bidirectional long short‐term memory (Bi‐LSTM), WaveNet encoders, and MIL frameworks to capture complex patterns associated with m^6^A modifications. A further increase in resolution is exemplified by tools such as Pum6a, SingleMod, and m6Aiso, which introduce single‐molecule sensitivity, robust quantification, and label‐efficient or semi‐supervised training strategies, which are critical for accurate detection under low stoichiometry or limited read coverage. Additionally, platforms such as m6ABasecaller and DEMINERS represent a new generation of modification‐aware base‐calling and scalable multiplexed analysis pipelines. These frameworks integrate m^6^A detection directly into real‐time base‐calling workflows and support high‐throughput applications with improved cost‐efficiency and precision. Collectively, these computational innovations have transformed m^6^A profiling from motif‐constrained inference into a high‐resolution, quantitative, and context‐aware discipline, enabling broader applications in transcriptome biology, disease research, and regulatory RNA analysis.

### 3.2 Computational Methods for Detecting Other RNA Modifications

While m^6^A has received the most extensive attention in nanopore‐based epitranscriptomic research, a growing body of work has begun to address the detection of other RNA modifications using DRS technology (Figure 3). These include Ψ, Nm, m^1^A, 5moU, and adenosine‐to‐inosine (A‐to‐I) editing, each with distinct chemical properties and biological functions. Detecting such modifications poses unique challenges due to their subtle effects on ionic current signals and limited training data. Recent advances in computational methods, particularly those employing supervised and unsupervised ML, have enabled the development of models capable of profiling these modifications with high sensitivity as well as resolution, and in many cases, without the need for chemical derivatization, antibody enrichment, or genetic knockouts. This section highlights representative tools and strategies designed to expand the modification landscape accessible through DRS, underscoring their potential to uncover novel regulatory layers in RNA biology (**Table** **2**).

**Table 2:** Comparative summary of computational tools for profiling other RNA modifications from Nanopore DRS data.

Methods	RNA modifications	Computational algorithm	Detection features	Application	Year	Refs.
ModQuant	Ψ	GBC	Current signals, base‐calling errors	*H. sapiens*	2021	[103]
NanoPsu	Ψ	EXT	Base‐calling errors, alignment features	*H. sapiens*	2021	[104]
Penguin	Ψ	SVM, RF, NN	Current signals, alignment features	*H. sapiens*	2022	[105]
IndoC	Ψ	SVM	Current signals	*H. sapiens* *S. cerevisiae*	2022	[106]
Mod‐*p* ID	Ψ	Fisher's test	Current signals	*H. sapiens*	2024	[107]
NanoPsiPy	Ψ	Chi‐square test	Base‐calling errors, alignment features	*H. sapiens*	2024	[108]
m1a‐prediction	m^1^A	XGBoost	Current signals	*H. sapiens*	2024	[109]
Dinopore	A‐to‐I	CNN	Current signals, base‐calling errors	*H. sapiens* *M. musculus* *X. laevis*	2022	[110]
Nm‐Nano	Nm	XGBoost, RF	Current signals, alignment features	*H. sapiens*	2024	[111]
NanoML‐5moU	5moU	SVM, RF, XGBoost	Current signals	IVT RNA	2024	[112]

ModQuant is a supervised ML framework designed for accurate and quantitative profiling of Ψ modifications in mRNA using DRS data.^[^ ^103^ ^]^ Trained on synthetic RNA standards containing site‐specific Ψ modifications, ModQuant leverages a comprehensive set of 45 signal‐ and base‐calling‐derived features extracted from ±2 nucleotides surrounding each candidate site. The model demonstrates high predictive accuracy across a wide range of sequence contexts and was robustly validated in seven human cell lines. By coupling synthetic controls with interpretable feature engineering, ModQuant establishes a generalizable and quantitative pipeline for Ψ detection, offering a scalable strategy for epitranscriptomic analyses of native transcriptomes.

NanoPsu is an ML framework for the transcriptome‐wide detection of Ψ modifications from DRS data.^[^ ^104^ ^]^ It employs extremely randomized trees (ExtraTrees, EXT) classifier trained on native ribosomal RNA reads, labeled using Illumina bisulfite sequencing, to identify Ψ‐modified sites. Unlike signal‐based methods, NanoPsu relies solely on 12 engineered features derived from base‐calling error profiles, achieving high predictive performance (AUC ∼0.94). Notably, it enables single‐read stoichiometry estimation and inter‐site linkage analysis, offering insights into modification co‐occurrence patterns along individual transcripts. By eliminating the need for synthetic training controls and direct current signal modeling, NanoPsu provides a quantitative, molecule‐resolved approach to Ψ profiling and represents a key innovation in DRS‐based epitranscriptomic analysis.

Penguin is an ML‐based tool developed for the prediction of Ψ sites from ONT DRS data in human cell lines.^[^ ^105^ ^]^ The framework integrates features derived from both raw nanopore signal data and base‐called *k*‐mers, enabling accurate Ψ site identification at single‐nucleotide resolution. Penguin achieved high predictive performance (>92% accuracy) across both random data splits and independent validation sets from the HEK293 and HeLa transcriptomes. By offering a comprehensive and scalable pipeline tailored to long‐read RNA data, Penguin addresses the limitations of earlier approaches confined to short‐read sequencing or non‐human models, thereby advancing Ψ detection in human transcriptomic studies.

IndoC is an informatics method for detecting Ψ modifications from nanopore DRS data and is designed to operate without the need for genetic knockouts or synthetic controls.^[^ ^106^ ^]^ The approach performs internal comparisons of trace‐derived values, ionic current intensities, and their statistical distributions between candidate sites and identical 5‐mer sequence contexts within the same dataset. These context‐aware features are integrated into a support vector machine (SVM) classifier to distinguish Ψ‐modified sites robustly from high‐mismatch or noisy positions. By eliminating external controls, IndoC offers a practical, scalable, and species‐agnostic strategy for Ψ mapping, facilitating broad application across diverse biological conditions and sample types.

Mod‐*p* ID is an innovative computational framework for transcriptome‐wide detection of Ψ modifications using DRS data, eliminating the need for chemical derivatization or cDNA conversion.^[^ ^107^ ^]^ Its core innovation lies in quantifying the Ψ occupancy across cell types by directly analyzing native RNA ionic current signatures. Mod‐*p* ID compares U‐to‐C base‐calling error rates between native and in vitro transcribed RNA using Fisher's exact test, supplemented by motif analysis to increase site‐level confidence. This approach enables robust, comparative Ψ profiling of native transcriptomes, offering a streamlined and scalable strategy for high‐resolution epitranscriptomic analysis.

NanoPsiPy is a computational tool for the identification and quantification of Ψ modifications from nanopore DRS data, based on statistical analysis of U‐to‐C base‐calling error rates.^[^ ^108^ ^]^ NanoPsiPy enables direct, stoichiometric mapping of Ψ sites in native human transcriptomes without the need for additional treatments. Beyond site‐level detection, this tool uncovers functional interplay between Ψ and other RNA modifications, providing new insights into epitranscriptomic cross‐talk. This streamlined, non‐invasive approach offers a scalable framework for transcriptome‐wide Ψ profiling, with potential applications in elucidating RNA modification dynamics in health and disease.

m^1^A‐prediction is the first dedicated computational workflow for the quantitative profiling of m^1^A modifications using DRS data.^[^ ^109^ ^]^ The method extracts electrical signal features such as the mean current, standard deviation, and event duration, and applies machine learning models, including XGBoost, trained on in vitro transcribed RNA datasets to predict m^1^A sites at single‐molecule resolution. Validation via binomial testing and downstream functional analyses in HEK293 cells revealed putative roles for m^1^A in RNA splicing and neurodegenerative pathways. While the tool represents a significant advance in DRS‐based m^1^A detection, its reliance on synthetic training data may constrain generalizability across diverse biological contexts.

Dinopore is a DL‐based framework for transcriptome‐wide identification of A‐to‐I RNA editing sites from DRS data.^[^ ^110^ ^]^ Unlike traditional alignment‐dependent methods, which are often confounded by SNPs and mapping artifacts, Dinopore directly models raw ionic current signals, base‐calling errors, and quality metrics using a multi‐dimensional CNN. This architecture enables accurate, single‐nucleotide resolution detection of inosine without requiring matched DNA sequencing or prior genome annotations. When trained on datasets from wild‐type and *ADAR1*‐knockout human stem cells and validated across multiple species, Dinopore achieves high predictive performance (AUC up to 0.98) and robust cross‐species generalizability. This approach represents a major methodological innovation, offering a robust, annotation‐free solution for RNA editing analysis and broadening the functional utility of nanopore DRS in epitranscriptomics.

Nm‐Nano is an ML‐based framework for the transcriptome‐wide detection of Nm sites from nanopore DRS data at single‐molecule resolution.^[^ ^111^ ^]^ The method integrates XGBoost and RF classifiers trained on signal‐level features and *k*‐mer embeddings, achieving high predictive accuracy (AUC up to 0.99) in human cell lines. By leveraging long‐read sequencing and advanced ML techniques, Nm‐Nano enables direct, high‐resolution mapping of Nm modifications, highlighting the potential of DRS‐based approaches for exploring Nm dynamics across diverse biological contexts.

NanoML‐5moU is an ML‐based framework for read‐level detection of 5moU modifications from nanopore DRS data.^[^ ^112^ ^]^ By extracting signal‐derived features including mean, median, standard deviation, and dwell time from *k*‐mers centered on U or 5moU residues, the method achieves single‐read resolution. Leveraging classifiers such as XGBoost, NanoML‐5moU attains high predictive accuracy (AUC up to 0.9567) without requiring matched control samples. This represents a methodological advancement in DRS‐based RNA modification detection, enabling *de novo* identification of 5moU and providing a valuable tool for quality control in synthetic mRNA therapeutics.

These emerging tools leverage a range of machine learning strategies trained on synthetic standards, native reads, or paired control datasets to enable RNA modification profiling at both single‐nucleotide and single‐molecule resolution. Supervised learning frameworks such as ModQuant, NanoPsu, and Penguin have demonstrated high accuracy for Ψ detection by utilizing signal‐derived or base‐calling error features. In parallel, label‐free approaches including IndoC, Mod‐*p* ID, and NanoPsiPy exploit intrinsic current distortions and base‐calling discrepancies to identify Ψ sites without the need for knockout models or synthetic controls. Expanding beyond Ψ, Chen et al. developed a workflow for m^1^A detection using XGBoost applied to signal‐based features, while Dinopore employs a deep convolutional neural network to detect A‐to‐I editing directly from raw ionic signals, circumventing genome alignment. Other specialized frameworks, such as Nm‐Nano and NanoML‐5moU, target Nm and 5moU, respectively, using ensemble learning models (e.g., XGBoost, RF) to achieve stoichiometric, high‐resolution detection. Collectively, these advances mark the maturation of nanopore DRS‐based epitranscriptomic profiling, offering scalable, quantitative, and modification‐specific alternatives to conventional chemical‐ or antibody‐based assays.

### 3.3 Computational Frameworks for Simultaneous Detection of Multiple RNA Modifications

As our understanding of the epitranscriptome deepens, it is increasingly evident that RNA modifications do not act in isolation but often interact in complex, combinatorial patterns to regulate transcript fate and function. This emerging concept of modification cross‐talk, where one modification influences the presence, function, or interpretation of another, has motivated the development of computational frameworks capable of simultaneously detecting multiple RNA modifications based on nanopore DRS data (Figure 3).

Unlike traditional single‐modification tools, these integrative approaches leverage the rich, multidimensional signal space of DRS and apply multi‐label learning or statistical modeling to resolve overlapping or co‐occurring modification signatures (**Table** **3**). By capturing co‐modification pattern interactions, these frameworks enable a more holistic view of transcript regulation and open new avenues for studying the layered architecture of the epitranscriptome. This section highlights recent efforts toward joint detection strategies and their potential to uncover mechanistic insights into RNA modification networks in health and disease.

**Table 3:** Computational tools for multi‐type RNA modification detection using Nanopore DRS data.

Methods	RNA modifications	Computational algorithm	Detection features	Application	Year	Refs.
TandemMod	m^6^A, m^5^C, m^1^A, hm^5^C, m^7^G, Ψ, I	CNN+BiLSTM	Current signals, base‐calling errors	*H. sapiens* *S. cerevisiae* *O. sativa*	2024	[30]
xPore	all	GMM	Current signals	*H. sapiens*	2021	[81]
Nanocompore	m^6^A, m^5^C, m^1^G, Ψ, I, 2′‐OMeA, m^7^G, m^6, 2^A	GMM	Current signals	*H. sapiens* *S. cerevisiae* *E. coli*	2021	[22]
nanoDoc	m^6^A, Ψ, Nm, m^5^C, etc.	CNN+DOC	Current signals	*E. coli* *SARS‐Cov2* *S. cerevisiae*	2021	[117]
nanoRMS	Ψ, Nm	KNNs	Current signals, base‐calling errors	*S. cerevisiae*	2021	[118]
Sequoia	m^6^A, m^5^C, etc.	DTW	Current signals, alignment features	*H. sapiens*	2021	[119]
JACUSA2	m^6^A, Ψ, etc.	NMF	Alignment features	*H. sapiens* *M. musculus*	2022	[120]
NanoSPA	Ψ, Nm	FNNs	Current signals	*H. sapiens*	2024	[121]
ELIGOS	m^6^A, m^1^A, 5moU, psU, m^7^G, Ino, hm^5^C, f^5^C	Fisher's test	Base‐calling errors	*H. sapiens*; *M. musculus*	2021	[122]
NMP classifier	m^6^A, m^5^C, Ψ, m^7^G, m^1^A	Linear SVM	Current signals	IVT RNA *S. cerevisiae*	2022	[123]
CHEUI	m^6^A, m^5^C	MIL+CNN	Current signals	*H. sapiens* *M. musculus*	2024	[124]
NA	m^6^A, m^5^C, hm^5^C, ac^4^C, Ψ, m^1^Ψ, m5U	CTC‐CRF+flip‐flop	Current signals	*H. sapiens* IVT RNA	2024	[125]
NanoMUD	Ψ, m^1^Ψ	BiLSTM	Current signals	IVT RNA *S. cerevisiae*	2024	[126]
iForest+	C‐to‐U	IF	Base‐calling errors	*H. sapiens*; *M. musculus*	2024	[127]
IL‐AD	m^6^A, m^1^A, m^5^C	IL+AD	Current signals	*H. sapiens* *E. coli* *M. musculus* *S. cerevisiae*	2024	[128]
DRAP3R	m^6^A, Ψ	–	Base‐calling errors	*H. sapiens*	2025	[129]
Uncalled4	m^6^A, m^5^C	bcDTW	Current signals	*H. sapiens*	2025	[130]
modCnet	ac^4^C, m^5^C	CNN+ BiLSTM	Current signals	*H. sapiens*; *S. cerevisiae*	2025	[131]
Dorado	Nm, Ψ, m^6^A, m^5^C, I	–	Base‐calling errors	–	2025	[113]
Remora	m^6^A, Ψ, I	–	Base‐calling errors	–	2025	[115]

ONT has recently expanded official support for RNA modification detection within its basecalling and analysis ecosystem. The Dorado basecaller includes pretrained models for the detection of multiple RNA modifications, including m^6^A, Ψ, m^5^C, inosine, and Nm. These pretrained models are compatible with the updated RNA004 chemistry and the rna004_130bps_sup@v5.2.0 model, enabling direct, per‐read identification of modifications from native ionic current signals.^[^ ^113^ ^]^ To streamline analysis, ONT also provides a modkit, a toolkit that parses modification calls embedded in the basecalled output.^[^ ^114^ ^]^ In parallel, ONT developed Remora, a lightweight inference engine designed to decouple modification calling from canonical basecalling. Remora operates as a secondary pass following initial basecalling, offering targeted detection of m^6^A, Ψ, and inosine with minimal computational overhead. This modular design allows for independent development and deployment of modification models, increasing flexibility and facilitating updates without retraining the basecaller.^[^ ^115^ ^]^ Together, Dorado and Remora represent an integrated framework for high‐throughput, native RNA modification profiling using nanopore direct RNA sequencing.

TandemMod is a transferable DL framework designed for the simultaneous identification of multiple RNA modification types from single‐sample nanopore DRS data at single‐nucleotide resolution.^[^ ^116^ ^]^ Leveraging in vitro epitranscriptome datasets with diverse sequence contexts and high‐confidence labels, the authors trained models to detect m^6^A, m^5^C, and m^1^A modifications, and applied transfer learning to extend detection capabilities to hm^5^C, m^7^G, Ψ, and inosine. TandemMod demonstrated robust accuracy across synthetic, human, and plant datasets, underscoring its broad applicability and generalizability. Validation through complementary molecular assays, including MeRIP‐seq, miCLIP, and m6ACE‐seq, confirmed strong concordance between predicted and experimentally supported modification sites. This integrative approach exemplifies the potential of deep learning frameworks to comprehensively profile diverse epitranscriptomic marks from complex biological samples.

xPore is a statistical framework for detecting differential RNA modifications from nanopore DRS data without requiring an unmodified control.^[^ ^81^ ^]^ By modeling per‐site ionic current distributions as two‐component Gaussian mixtures, xPore estimates modification rates using variational Bayesian inference, enabling quantitative and site‐specific detection. The method was validated using METTL3 knockout and knockdown in HEK293T cells, with orthogonal support from multiple experimental platforms, including m6ACE‐seq, miCLIP, MAZTER‐seq, and RNA mixing assays. Compared with both supervised (e.g., EpiNano) and unsupervised (e.g., Tombo, Nanocompore) approaches, xPore demonstrated superior precision, recall, and computational efficiency. Its applicability has been further demonstrated across diverse biological contexts, including human cancer cell lines and clinical multiple myeloma samples, highlighting its robustness and utility for modification profiling from limited or heterogeneous RNA inputs.

nanoDoc is a DL framework designed to detect a wide spectrum of RNA modifications from DRS data using raw ionic current signals.^[^ ^117^ ^]^ Employing a CNN in combination with deep one‐class (DOC) classification, the nanoDoc is trained exclusively on unmodified synthetic sequences and identifies modifications by quantifying deviations from the learned baseline signal. Without requiring labeled modified data or prior knowledge of modification types, the method achieves high performance (AUC = 0.96) across 23 different RNA modifications. Its reference‐free, unsupervised nature allows broad applicability, and the framework has been successfully applied to rRNA, mRNA, and viral transcriptomes, positioning nanoDoc as a versatile tool for *de novo* epitranscriptomic profiling.

Nanocompore is a model‐free comparative framework for detecting differential RNA modifications from DRS data.^[^ ^22^ ^]^ It identifies modification‐associated signal changes by statistically comparing the ionic current intensity and dwell time distributions between two experimental conditions. When validated on synthetic RNAs bearing seven distinct modifications and on biological samples, including yeast and METTL3‐knockdown human cells, Nanocompore has demonstrated robust performance across diverse contexts. Orthogonal validation using miCLIP and meRIP‐qPCR further supports its reliability. By avoiding assumptions about specific modification types or sequence motifs, Nanocompore provides a flexible and broadly applicable tool for differential RNA modification analysis.

nanoRMS is a single‐molecule analysis framework designed to quantify RNA modification stoichiometry from DRS data.^[^ ^118^ ^]^ It integrates multiple nanopore‐derived signal features, including ionic current intensity, dwell time, and base probability to enable both supervised and unsupervised detection of modifications such as Ψ and Nm. The method was validated using yeast snoRNA knockout strains and benchmarked against nanoCMC‐seq, demonstrating its capacity to detect and quantify non‐m^6^A RNA modifications with high resolution and flexibility. By providing modification stoichiometry at the per‐read level, nanoRMS offers a valuable tool for quantitative epitranscriptomic profiling beyond canonical marks.

Sequoia is an interactive visual analytics platform designed to explore and distinguish RNA modification signals in DRS data.^[^ ^119^ ^]^ By integrating dynamic time warping (DTW) with t‐distributed stochastic neighbor embedding (t‐SNE), Sequoia enables unsupervised clustering of raw ionic current signals, facilitating the identification of RNA modifications such as m^6^A and m^5^C in HeLa cells. The platform incorporates a multi‐view visualization framework, including boxplots, signal traces, and t‐SNE plots with adjustable parameters (e.g., DTW penalty), allowing users to iteratively refine clustering and detect qualitative signal differences between modified and unmodified reads. Sequoia represents a novel computational approach that complements predictive models by offering intuitive, hypothesis‐generating insights into RNA modification‐associated signal features, and may serve as a foundational tool for the future development of automated and interpretable epitranscriptomic analysis methods.

JACUSA2 is a versatile, platform‐agnostic computational framework for detecting diverse RNA modifications by analyzing base substitution, insertion, deletion, and reverse transcription arrest events across sequencing data.^[^ ^120^ ^]^ Compared with both the Illumina and Oxford Nanopore platforms, JACUSA2 enables the detection of modifications such as m^6^A and Ψ through statistical modeling and unsupervised learning, without relying on raw signal‐level data. A notable innovation is its application of non‐negative matrix factorization (NMF) to deconvolute complex modification‐associated patterns from alignment‐derived features, facilitating robust modification calling across experimental conditions. By abstracting modification signals into a platform‐independent representation, JACUSA2 provides a flexible and scalable solution for comparative epitranscriptomic analyses across datasets and sequencing technologies.

NanoSPA is a DL‐based framework designed for the simultaneous detection of m^6^A and Ψ from DRS data.^[^ ^121^ ^]^ The method employs motif‐specific feedforward neural networks trained on orthogonally validated m6A‐SAC‐seq datasets, integrating features from the raw ionic current, dwell time, and base probability (trace) to capture modification‐specific signatures. Through extensive validation across multiple perturbation experiments and external benchmark datasets, NanoSPA demonstrates high accuracy in the joint profiling of two chemically distinct modifications in human transcriptomes. By enabling concurrent analysis of m^6^A and Ψ from a single dataset, NanoSPA provides an important tool for investigating modification co‐occurrence, interplay, and potential cross‐talk in the epitranscriptome.

ELIGOS is a computational framework for detecting RNA modifications from nanopore direct RNA sequencing by leveraging systematic base‐calling error profiles between native RNA reads and control sequences such as in vitro transcribed or cDNA references.^[^ ^122^ ^]^ Rather than relying on ML or pre‐labeled training data, ELIGOS employs statistical modeling of error signatures, termed reference‐based error models (rBEMs) to infer the presence of modified nucleotides based on recurring signal disruptions. This approach enables the detection of multiple RNA modifications, including m^6^A, m^1^A, 5moU, and Ψ at single‐base resolution across synthetic and endogenous RNA samples. While the method excels in its label‐free and reference‐agnostic design, it lacks the ability to resolve specific modification types and performs suboptimally for certain marks such as m^5^C, highlighting trade‐offs between generalizability and biochemical specificity.

The NMP classifier represents a novel enzymatic‐nanopore strategy for RNA modification analysis that departs fundamentally from DRS paradigms.^[^ ^123^ ^]^ In this method, RNA molecules are enzymatically hydrolyzed into individual nucleoside monophosphates (NMPs), which are then analyzed using a PBA‐modified MspA nanopore. Each NMP generates distinct current blockade and noise profiles, enabling accurate classification of a broad spectrum of RNA modifications at the mononucleotide level. Unlike DRS‐based tools that infer modifications within intact transcripts, the NMP classifier offers direct biochemical identification of RNA modifications, albeit without positional or sequence context. This approach underscores a powerful orthogonal strategy for comprehensive epitranscriptomic profiling, which is particularly suited for validating modification identities and expanding the catalog of detectable RNA marks.

CHEUI is a DL‐based framework that enables simultaneous, transcriptome‐wide detection of m^6^A and m^5^C modifications from DRS data at single‐molecule resolution, without the need for control samples.^[^ ^124^ ^]^ The method leverages a two‐stage CNN architecture to first classify individual reads as modified or unmodified and then aggregate predictions to infer site‐level stoichiometry. By directly modeling raw signal features, CHEUI overcomes the limitations of motif dependence and modification specificity that constrain many earlier tools. Importantly, CHEUI allows joint profiling of multiple epitranscriptomic marks within the same RNA molecule, revealing co‐occurrence and potential cross‐talk between m^6^A and m^5^C in human transcripts. This ability to detect combinatorial modification patterns represents a significant advance toward integrative, context‐aware epitranscriptomic analysis.

Diensthuber et al. introduced two supervised DL‐based base‐calling models for nanopore DRS data, which were designed to enhance RNA modification detection through improved base‐calling accuracy.^[^ ^125^ ^]^ The first model utilized a flip‐flop neural network architecture trained on unmodified in vitro transcribed RNA, while the second employed a high‐accuracy model based on connectionist temporal classification with conditional random fields (CTC‐CRF). These models were shown to amplify base‐calling error signatures at known modification sites, including m^6^A, m^5^C, hm^5^C, ac^4^C, Ψ, m^1^Ψ, and m^5^U, thereby increasing the sensitivity of downstream detection tools such as ELIGOS2. This work underscores the critical role of the basecaller architecture in shaping the interpretability of modification signals, and highlights a new direction for epitranscriptomic method development: optimizing base‐calling itself as a modifiability‐aware analytical step.

NanoMUD is a DL framework that enables simultaneous detection of Ψ and m^1^Ψ from DRS data at single‐molecule resolution.^[^ ^126^ ^]^ The method employs bidirectional long short‐term memory (BiLSTM) neural networks trained on synthetic, motif‐specific RNA sequences, capturing nuanced signal features to differentiate between two chemically similar modifications. NanoMUD integrates a regression‐based stoichiometry estimation module, allowing quantitative profiling of modification levels across transcripts. In terms of exceptional accuracy (AUC up to 0.998), NanoMUD represents the first approach capable of distinguishing Ψ from m^1^Ψ directly from raw current signals, providing a powerful tool for studying both native RNA biology and synthetic RNA therapeutics, where m1Ψ is commonly used to increase mRNA stability and translation.

iForest+ is a novel ML‐based method designed for the accurate detection of C‐to‐U RNA editing events from DRS data.^[^ ^127^ ^]^ Leveraging the Isolation Forest (IF) algorithm, iForest+ models genuine editing sites as statistical anomalies within basecalling‐derived features such as base quality, mismatches, and indels. This approach distinguishes true editing events from systematic nanopore sequencing errors without relying on raw current signals or matched knockout controls. When validated across synthetic constructs, mouse macrophage cells, and engineered human HEK293T lines, iForest+ demonstrates high precision, efficiency, and generalizability, offering a fast and lightweight tool for robust RNA editing profiling in diverse biological and biomedical contexts.

IL‐AD is an innovative computational tool developed for detecting RNA modifications such as m^6^A, m^1^A, and m^5^C from DRS data at single‐nucleotide and single‐molecule resolutions.^[^ ^128^ ^]^ It uniquely combines incremental learning to refine base‐calling accuracy on modification‐rich reads with anomaly detection to identify signal deviations associated with RNA modifications. This approach allows for sequence‐context‐free and cross‐species detection, enhancing both the accuracy and generalizability of RNA modification profiling and marking a significant advancement in epitranscriptomic research.

DRAP3R is not a novel computational model but rather an integrative framework that combines existing nanopore tools to detect m^6^A and Ψ modifications using base‐called probabilities.^[^ ^129^ ^]^ Its key innovation lies in its experimental design, particularly the use of a custom poly(U)‐targeting adapter that selectively sequences Pol III transcribed RNAs. This approach facilitates the study of co‐ and post‐transcriptional RNA modifications in noncoding transcripts that have been largely overlooked, expanding the scope of epitranscriptomic analysis beyond conventional mRNA targets.

Uncalled4 is an advanced signal‐level alignment toolkit designed to improve RNA modification detection from DRS data.^[^ ^130^ ^]^ It introduces a basecaller‐guided dynamic time warping (bcDTW) algorithm alongside a compact BAM‐based data structure to efficiently align and encode per‐position ionic current features such as the mean, standard deviation, and dwell time. Uncalled4 enhances m^6^A detection sensitivity when integrated with tools like m6Anet, and supports m^5^C detection through a KS‐statistics‐based approach. Compared with the latest Oxford Nanopore Technologies chemistries, Uncalled4 outperforms existing methods, identifying ≈26% more m^6^A sites than Nanopolish does in human cell line datasets, thus representing a significant step forward in high‐resolution epitranscriptomic profiling.

modCnet is a DL framework for the simultaneous detection of ac^4^C and m^5^C RNA modifications from DRS data.^[^ ^131^ ^]^ It combines a CNN, bidirectional long short‐term memory networks (biLSTMs), and attention layers to analyze both raw ionic current signals and base‐level statistical features. This integrated approach enables accurate, single‐molecule resolution profiling of cytidine modifications, facilitating insights into their distribution and co‐occurrence within transcriptomes.

Recent advances in nanopore‐based technologies have enabled the simultaneous detection of multiple RNA modifications, overcoming key limitations of traditional enrichment‐based or single‐modification assays. These approaches leverage diverse strategies from deep learning models such as TandemMod, nanoDoc, CHEUI, modCnet, and NanoMUD, to statistical frameworks like xPore, Nanocompore, and ELIGOS, as well as basecaller‐level innovations exemplified by Uncalled4, Diensthuber et al., and m6Abasecaller to jointly profile RNA modifications including m^6^A, m^5^C, Ψ, m^1^A, hm^5^C, and ac^4^C at single‐base or single‐molecule resolution. While some tools (e.g., TandemMod, CHEUI) utilize supervised learning trained on synthetic or control datasets, others (e.g., nanoDoc, Nanocompore) adopt unsupervised or comparative approaches that detect modifications without requiring labeled data. Methods such as iForest+ and IL‐AD extend this landscape by leveraging error profiles or signal deviations in native reads to enable modification‐aware detection even in the absence of control samples. Importantly, these frameworks provide critical insights into the co‐occurrence and cross‐talk between different RNA modifications within individual RNA molecules, allowing researchers to dissect complex epitranscriptomic regulation and interplay at unprecedented resolution. Collectively, these advances not only facilitate accurate stoichiometry estimation and co‐modification analysis but also exhibit robust cross‐sample and cross‐species generalizability, significantly advancing the resolution and scope of epitranscriptomic profiling with nanopore direct RNA sequencing data.

## 4 Challenges and Opportunities in Nanopore‐Based RNA Modification Detection

Despite rapid advances in nanopore DRS technologies and computational algorithms, several technical challenges remain in accurately detecting RNA modifications. These include the low abundance of certain modifications, signal overlap from adjacent or co‐occurring marks, and the complexity of interpreting raw ionic current data. Distinguishing true modification‐induced signal deviations from natural noise is especially difficult in regions with high structural variability or limited read coverage. Additionally, the scarcity of dedicated training datasets and orthogonal validation methods for many modifications limits model generalizability and detection accuracy.

To overcome these challenges, recent studies have explored novel strategies to increase both sensitivity and specificity, such as positive‐unlabeled learning, MIL, and semi‐supervised frameworks that operate without the need for knockout controls or synthetic standards. Signal deconvolution and motif‐aware modeling further help resolve overlapping modification signals. These innovations open new avenues for investigating underexplored modifications, including Nm, ac^4^C, and inosine, and enable the development of robust, scalable tools for transcriptome‐wide, isoform‐resolved, and single‐molecule epitranscriptomic analyses.

The integration of ONT DRS with advanced computational methods has yielded critical biological insights into RNA modification distribution, dynamics, and function. Isoform‐specific m^6^A profiling has uncovered novel roles in alternative splicing, mRNA localization, and translation efficiency. Tools like CHEUI and TandemMod have facilitated simultaneous detection of multiple modifications (e.g., m^6^A and m^5^C) within individual transcripts, revealing co‐regulatory networks and cross‐talk between distinct epitranscriptomic marks.

While numerous machine learning‐based algorithms have been developed for RNA modification detection, the absence of standardized evaluation frameworks has historically hindered objective comparison and reproducibility. The RMaP challenge addressed this gap by introducing the first benchmarking framework for m^6^A, m^5^C, and Ψ detection from nanopore DRS using synthetic RNA datasets with known modifications.^[^ ^132^ ^]^ This initiative established critical standards for methodological transparency, data consistency, and future computational tool development.

Beyond model eukaryotes, recent advances have extended to non‐model organisms, uncovering new biological layers across species. For example, Tan et al. combined optimized DRS protocols with computational pipelines to generate high‐resolution m^6^A maps in *E. coli*, providing one of the first comprehensive views of prokaryotic RNA modification.^[^ ^133^ ^]^ This work underscores the potential of DRS‐based epitranscriptomics for studying bacterial gene regulation, microbial‐host interactions, and RNA‐based diagnostics in infectious diseases.

The nanom6A algorithm was applied to study m^6^A dynamics in *Glycine max* under salt stress.^[^ ^134^ ^]^ Using ONT DRS, over 49000 m^6^A sites were identified across 8200 genes in salt‐ and mock‐treated leaf and root samples. Salt treatment led to substantial remodeling of the m^6^A landscape, particularly in roots, where more than 8700 unique m^6^A sites were gained. Gene Ontology analysis revealed enrichment of salt‐responsive genes, suggesting a role for m^6^A in stress adaptation.

Additional examples including CHEUI in acute myeloid leukemia (AML), further illustrate the clinical potential of this technology. By applying ONT DRS with CHEUI, Chen et al. have detected widespread RNA hypermethylation of m^5^C in bone marrow samples from AML patients compared with samples taken during complete remission (CR).^[^ ^135^ ^]^ Principal component analysis of m^5^C profiles revealed distinct clustering between the AML and CR states, with AML samples showing broader dispersion. m^5^C‐modified genes in the AML group were significantly enriched in myeloid cell activation pathways (e.g., leukocyte adhesion, proliferation, and cytokine production), suggesting a functional role in disease progression. Conversely, the CR samples showed reduced m^5^C levels and were enriched for cellular stress and immune response pathways. These findings suggest that m^5^C methylation patterns captured by ONT DRS may serve as dynamic biomarkers for disease state and therapeutic response in hematological malignancies.

Further, two studies demonstrate the power of ONT DRS combined with xPore to resolve RNA methylation dynamics in *Arabidopsis*.^[^ ^136^, ^137^ ^]^ First, xPore was used to examine how the m^6^A reader ECT8 mediates mRNA localization into stress granules (SGs) during abscisic acid (ABA) treatment. mRNAs enriched in SGs from wild‐type seedlings showed significantly higher m^6^A levels than in the ect8‐1 mutant. xPore identified over 7600 differentially methylated sites located near stop codons and 3′ UTRs, and was associated with stress and metabolic processes. These findings indicate that ECT8 selectively recruits m^6^A‐marked RNAs into SGs in response to ABA. In a complementary study, ONT DRS and xPore were used to investigate the plant‐specific m^6^A writer FIO1. A comparison between wild‐type and fio1‐2 seedlings revealed 3459 hypomethylated sites in 2068 protein‐coding genes. FIO1‐dependent sites were enriched in coding sequences, peaking before stop codons, highlighting a distinct m^6^A deposition pattern from that of canonical writers like FIP37 and VIR. These studies collectively demonstrate how ONT DRS and xPore can dissect spatial and condition‐specific m^6^A dynamics and link them to specific protein regulators and functional outcomes.

Collectively, these advances demonstrate the broad utility and biomedical relevance of nanopore‐based RNA modification detection. As these approaches continue to mature, they are poised to drive discoveries in fundamental biology and clinical research, reshaping our understanding of RNA function in both biology and disease.

## 5 Conclusion and Future Perspectives

The advent of ONT DRS has revolutionized epitranscriptomics, enabling direct, real‐time, and amplification‐free detection of RNA modifications at single‐molecule resolution. When coupled with ML frameworks, ONT DRS has facilitated high‐resolution mapping of diverse RNA marks, including m^6^A, m^5^C, Ψ, m^1^A, and others, shedding light on post‐transcriptional gene regulation across cell types, conditions, and species.

Despite these advances, several key challenges persist. A major bottleneck lies in accurately resolving adjacent or co‐occurring modifications, where overlapping signal distortions can obscure individual modification identities. Signal variation due to RNA structure, coverage bias, or sample preparation artifacts also complicates interpretation. Cross‐sample and cross‐species model transferability remains limited, especially when applying trained models to non‐canonical modifications or data from underrepresented organisms. Furthermore, the scarcity of standardized, high‐quality training datasets and gold‐standard validation benchmarks hinders reproducibility and limits broad adoption.

Addressing these issues will require the development of next‐generation computational and experimental tools. Modification‐aware basecallers (e.g., m6ABasecaller, Densecall), multi‐modification frameworks (e.g., CHEUI, TandemMod), and algorithms that integrate ionic current signals, sequence contexts, and structural features are pivotal. Equally important is the expansion of diverse, high‐quality training datasets including biological replicates, synthetic constructs, and data from non‐model species to support the development of robust and transferable algorithms.

Looking ahead, integrating ONT DRS with multi‐omics platforms such as proteomics, metabolomics, ATAC‐seq, and chromatin conformation assays will help uncover how RNA modifications interface with broader regulatory networks. Combining modification maps with spatial transcriptomics, single‐cell sequencing, or ribosome profiling could illuminate previously inaccessible layers of RNA regulation, including cell‐type‐specific and subcellular localization‐dependent effects.

Finally, biomedical applications of RNA modification profiling are rapidly emerging. From cancer epitranscriptomics to microbial diagnostics and therapeutic RNA design, nanopore‐based modification profiling is poised to drive both mechanistic discoveries and clinical innovation. To fully realize this potential, future work must prioritize algorithmic refinement, rigorous benchmarking, and experimental validation of the predicted modifications and their functional consequences.

In summary, ONT DRS has opened a new era in RNA biology in which the dynamic and multilayered nature of the epitranscriptome can be decoded at unprecedented scale and resolution. Continued innovation will be essential to fully chart this landscape and translate it into meaningful biological and biomedical insights.

## Conflict of Interest

The authors declare no conflict of interest.

## Data Availability

All data that support this study are publicly available in the cited sources.
